# Native Electrospray Mass Spectrometry of DNA G-Quadruplexes in Potassium Solution

**DOI:** 10.1007/s13361-014-0890-3

**Published:** 2014-04-30

**Authors:** Adrien Marchand, Valerie Gabelica

**Affiliations:** 1 University Bordeaux, IECB, ARNA Laboratory, F-33600 Pessac, France; 2 INSERM, U869, ARNA Laboratory, F-33000 Bordeaux, France

**Keywords:** DNA structures, G‐quadruplexes, Native, Mass spectrometry, Ion mobility, Potassium, Trimethylammonium, Adducts, Cation binding

## Abstract

A commonly used electrolyte in electrospray mass spectrometry (ESI-MS) of biomolecules is ammonium acetate (NH_4_OAc). Although some nucleic acid structures such as duplexes require only proper physiological ionic strength (whatever the monovalent ions) to be properly folded in ESI-MS conditions, the folding of some other nucleic acid structures such as DNA G-quadruplexes also depends on direct binding of specific cations. Here, we developed ESI-MS compatible conditions that allow one to observe DNA G-quaduplexes with K^+^ ions specifically bound between G-quartets. NH_4_OAc was replaced with trimethylammonium acetate (TMAA), at concentrations up to 150 mM to provide physiological ionic strength, and the solution was doped with KCl at concentrations up to 1 mM. The trimethylammonium ion is too large to coordinate between G-quartets, where only K^+^ ions bind. Compared with the equivalent NH_4_OAc/KCl mixtures, the TMAA/KCl mixtures provide cleaner spectra by suppressing the nonspecific adducts, and favor the formation of similar stacking arrangements as in 100 mM KCl (physiologically relevant cation) for the polymorphic human telomeric DNA G-quadruplexes. This new sample preparation method can be exploited to determine the number of potassium binding sites in new sequences, to screen ligand binding to the structures favored in potassium, and to transfer potassium-bound G-quadruplexes to the mass spectrometer for gas-phase structural probing, as illustrated herein with ion mobility spectrometry experiments.

**Electronic supplementary material:**

The online version of this article (doi:10.1007/s13361-014-0890-3) contains supplementary material, which is available to authorized users.

**Figure:**
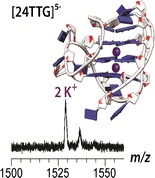
ᅟ

## Introduction

**Figure 1:**
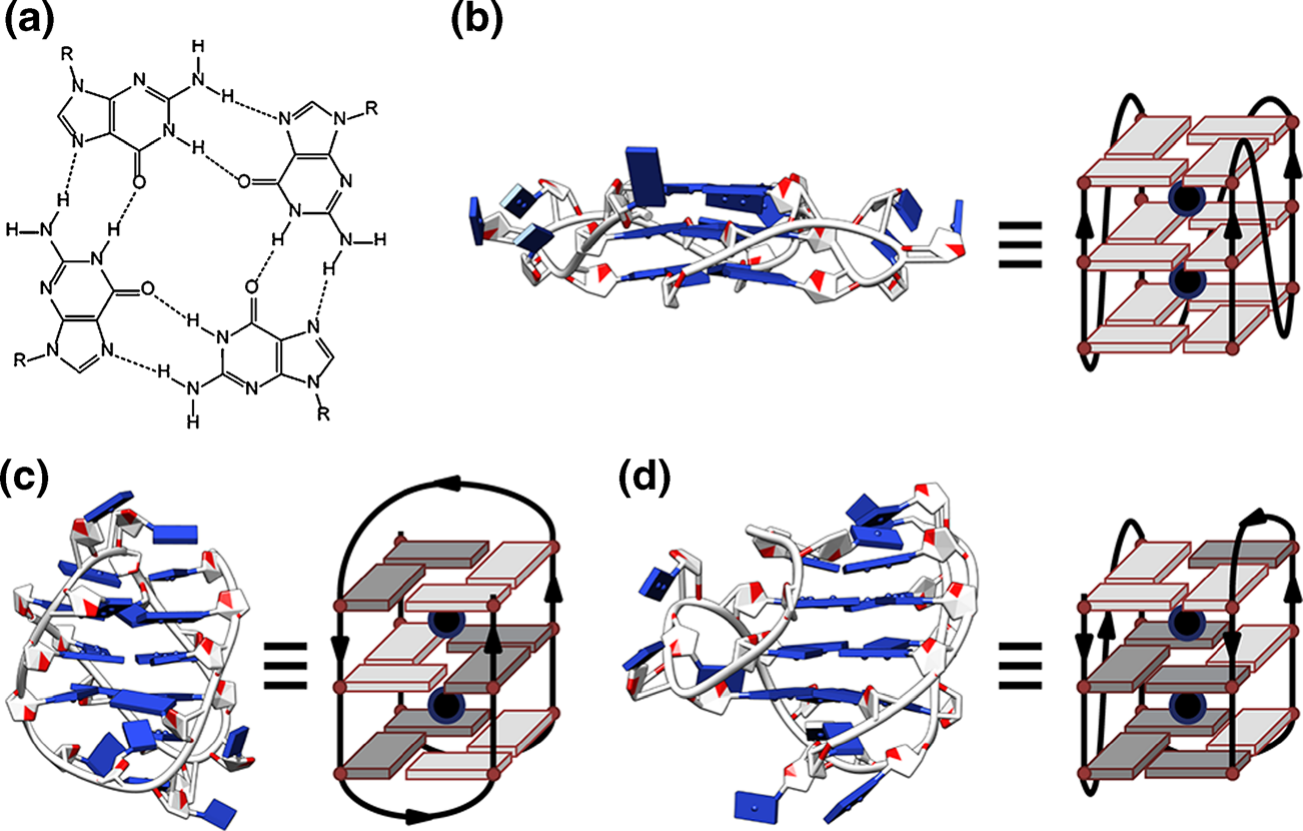
**(a)** A guanine quartet (G-quartet). **(b)** X-ray crystal structure of the parallel G-quadruplex form of d(A(GGGTTA)_3_GGG) in K^+^ conditions [12] (PDB ID: 1KF1). **(c)** NMR structure of the antiparallel G-quadruplex form of d(A(GGGTTA)_3_GGG) in Na^+^ solution [13] (PDB ID: 143D). **(d)** NMR structure of the hybrid G-quadruplex form of d(TT(GGGTTA)_3_GGGA) in K^+^ solution [15] (PDB ID: 2GKU). The schemes underline strand directionality, and base-sugar conformations (light grey for *anti*, dark grey for *syn*)

G-quadruplexes are non-classical DNA or RNA structures consisting of stacked guanine quartets (G-quartets) made by Hoogsteen hydrogen-bonding between guanines (Figure 1a). Potassium, sodium, or ammonium cations also stabilize the structure by coordinating to the O6 oxygen of guanines. Owing to their size, ammonium, and potassium ions are located in-between two G-quartets and, therefore, coordinate with eight O6 atoms, whereas smaller sodium ions can also access the center of G-quartets. G-quadruplexes can be found in vitro in the promoter regions of oncogenes [1–4] or in the human telomeric region [5]. Some recent work from Balasubramanian’s group showed that G-quadruplex DNA structures are present in human cells [6]. Because of their supposed role in cancer, G-quadruplexes are often studied as targets for anti-cancer drugs [7–10].

The most studied G-quadruplex forming sequence is probably the human telomeric sequence, consisting of (TAGGGT)_n_ repeats. Human telomeric G-quadruplexes present a high level of polymorphism in vitro depending on their core sequence, flanking bases, salt conditions, the nature of the sugar (DNA versus RNA),… [11]. For example, parallel G-quadruplexes have been reported by X-ray crystallography studies for d(A(GGGTTA)_3_GGG) in K^+^ conditions (Figure 1b) [12]. In contrast, by NMR in solution, an antiparallel basket-type form with three G-quartets was observed in Na^+^ conditions for d(A(GGGTTA)_3_GGG) (Figure 1c) [13], and one with only two G-quartets in K^+^ conditions for d((GGGTTA)_3_GGGT) [14]. In K^+^ solution, a hybrid form can also be adopted by sequences d(TT(GGGTTA)_3_GGGA) (Figure 1d) [15], d(TA(GGGTTA)_3_GGG) [16], or d(TA(GGGTTA)_3_GGGTT) [17].

Taking this high level of polymorphism into account is important to interpret experimental results obtained by different techniques, which sometimes impose using different solution conditions. G-quadruplexes are indeed often studied in different buffer conditions depending on the assay. Phosphate and Tris/HCl buffers are often used to obtain neutral pH, and are supplemented by sodium chloride or potassium chloride, usually at a concentration around 100 mM. When screening ligands towards G-quadruplexes, a sensible approach to acknowledge this polymorphism is to test ligand binding to different sequences and under a range of solution conditions. For example, ligand binding assays are often compared in Na^+^ and in K^+^ to probe whether a ligand has any preference for antiparallel forms (in Na^+^) or more parallel or hybrid forms (in K^+^). In indirect ligand binding assays such as changes in melting temperature, sometimes the K^+^ concentration is decreased to 10 or even 1 mM in order to destabilize the G-quadruplex, so as to be able to measure the effect of the ligand on the stability [18].

Mass spectrometry (MS)-based G-quadruplex drug binding assays offer several advantages compared with other traditional methods [19] because it allows direct stoichiometry observation and quantification of each peak [20]. Nevertheless, none of the buffers mentioned above are compatible with native electrospray ionization because they are nonvolatile. To bypass this limitation, ammonium acetate (NH_4_OAc) is used to fix the ionic strength. When G-quadruplex forming sequences are electrosprayed in NH_4_OAc, specific ammonium cations are retained in the complexes. They are able to coordinate between the G-quartets [21, 22]. G-quadruplex structures made in ammonium acetate solution are, however, less stable and more polymorphic than those formed in potassium chloride solution [20].

Spraying nucleic acids in the presence of physiological cations is an important challenge that has been addressed by several groups, either for G-quadruplexes (K^+^) or for RNA structures (Mg^2+^). Previously, Evans et al. [23] and the team of Brodbelt [24] reported tetramolecular G-quadruplexes electrosprayed with potassium adducts. In their respective works, the G-quadruplexes are preformed into potassium conditions before being partially desalted by ethanol precipitation or filtering, and resuspended in ammonium acetate for ESI-MS analysis. However, although this worked well for tetramolecular G-quadruplexes that are parallel-stranded whatever the cation, for polymorphic G-quadruplexes, ammonium acetate replacement will also displace the conformational equilibria. McLuckey’s group exploited ion–molecule reactions, and used vaporized acetic or formic acid inside the desolvation cell to displace metal counter-ions that stick to the phosphate groups [25]. This method allowed keeping native species in solution because the interaction with the acid is made in the gas phase and not in the solution phase. However, gas-phase structures may be disrupted. Fabris’ group exploited ion–ion reactions in FTICR-MS to clean up sodium and magnesium adducts from diverse DNA and RNA structures [26]. However these approaches are not practical on an everyday basis and require specialized instrumentation.

Here, we show that trimethylammonium acetate (TMAA) can advantageously replace ammonium acetate (NH_4_OAc) to carry out ESI-MS experiments on G-quadruplexes in the presence of K^+^. The key idea is that the trimethylammonium cation is too bulky to coordinate between quartets, and that only K^+^ can fill the inter-quartet sites. A similar approach was used by Gray and Chaires for solution spectroscopy-monitored G-quadruplex titrations by KCl in the presence of tetrabutylammonium phosphate [27]. Trialkylamines or trialkylammonium buffers are commonly used in ESI-MS. In 1994, Bischoff’s group compared the efficiency of triethylamine (TEA), trimethylamine (TMA), and NH_4_OAc to remove sodium adducts on oligomers from 12- to 132-mer oligonucleotides [28]. They showed that ammonium acetate is the least efficient in reducing the amount of adducts. Ostrander’s group [29] proposed the triethylammonium acetate (TEAA) additive to clean up their mass spectra from sodium and potassium adducts. In their case, they fixed the pH to 7 thanks to imidazole. Lemaire et al. [30] also reported that triethylammonium bicarbonate is useful to study proteins and proteins complexes under non-denaturing conditions. They showed a charge state displacement to lower charges induced by this additive, and were able to distinguish NADH adducts on the dimer made by two alcohol dehydrogenase. The present study shows for the first time the advantage of using TMAA for studying nucleic acids by electrospray mass spectrometry in native conditions (neutral pH, physiological ionic strength, and with sufficient amounts of KCl to form presumably native G-quadruplex structures).

## Experimental

### Materials

Oligonucleotides were purchased lyophilized and RP-cartridge purified from Eurogentec (Seraing, Belgium). In this work, the following nomenclature will be used: the hybrid G-quadruplex forming sequence d(TT(GGGTTA)_3_GGGA) = 24TTG; the hybrid G-quadruplex forming sequence d(TA(GGGTTA)_3_GGG) = 23TAG; a 22-mer control oligonucleotide with twelve guanines but incapable of forming a G-quadruplex: d(GGG-ATG-CGA-CAG-AGA-GGA-CGG-G) = 22non-G4; a tetramolecular parallel G-quadruplex made by four d(TGGGGT) strands = TG_4_T; and a bimolecular G-quadruplex made by two d(GGGGTTTTGGGG) strands = G_4_T_4_G_4_. Stock solutions were first prepared at 0.5 to 1 mM in water. The stock concentrations were determined by UV absorbance at 260 nm, with extinction coefficients calculated using Cavaluzzi-Borer Correction [31], and measured on an Agilent Cary 100 UV spectrophotometer. Water was nuclease-free grade from Ambion (Applied Biosystems, Lennik, Belgium). Ammonium acetate (NH_4_OAc, Ultra for Molecular Biology, Fluka), trimethylammonium acetate (TMAA, Ultra for UPLC, Fluka), potassium (KCl, >99.999%, Sigma) and sodium chloride (NaCl, >99.999%, Sigma) were purchased from Sigma-Aldrich (Saint-Quentin Fallavier, France). The solutions were prepared at room temperature (no annealing), 2 to 3 h before the circular dichroism (CD) or MS measurements were performed. Some measurements were repeated after 1 wk, and no change in CD or MS results was observed. The pH of TMAA and DNA-containing TMAA solutions was in the 6.7–6.8 range. TMAA solution was therefore always used as received without complementary pH-adjustment.

### Mass Spectrometry (MS)

All mass spectra were obtained in negative ion mode with injection concentrations of 5 μM in G-quadruplexes and 5 μM internal standard dT_6_. Native electrospray mass spectra of oligonucleotides were obtained using several instruments, with similar results. (1) On an LCT Premier mass spectrometer (Waters, Manchester, UK), the ESI source voltage was set to 2.2 kV with a desolvation temperature of 60°C. Unless otherwise mentioned, the ion guide 1 voltage is 50 V. The source pressure was increased to 35 mbar. This pressure is measured using a Center Two probe (Oerlikon Leybold Vacuum, Cologne, Germany). The syringe injection flow was fixed to 200 μL/h. In all experiment 5 μM dT_6_ (monoisotopic mass 1762.318 Da) was used as internal standard. (2) An Exactive ESI-Orbitrap mass spectrometer (Thermo Scientific, Bremen, Germany) was also used to monitor the titration of TG_4_T by KCl. In this case, ESI spray voltage and capillary voltage are 2.75 kV and –20 V, respectively. Capillary temperature is set to 150°C. Tube lens and skimmer voltage are fixed to –180 V and –10 V. In order to help the desolvation process to occur, the HCD cell voltage is set to 10 eV and the cell pressure is 2.5 × 10^–5^ mbar.

### Ion Mobility Spectrometry (IMS)

The collision cross sections of NH_4_^+^-containing and K^+^-containing G-quadruplexes were determined using a Synapt G2S mass spectrometer (Waters, Manchester, UK). The electrospray capillary was at 2.04 kV, the source and desolvation temperatures were 30°C and 40°C, respectively, and the sampling cone was at 28 V. The trap and transfer collision energies were 2 and 4 V, respectively. All these parameters ensured minimal fragmentation outside the IMS cell. The helium gas flow in the pre-IMS cell was 200 mL/min, and the nitrogen gas flow in the IMS cell was 90 mL/min. The IMS T-wave was operated at 40 V and 600 m/s. The CCS was calibrated following the procedure of Ruotolo et al. [32] using dT_6_^2–^ (306 Å^2^), dT_6_^3–^ (333 Å^2^), [dTG_4_T]_4_^4–^ (730 Å^2^), [dTG_4_T]_4_^5–^ (775 Å^2^) [33], and [dTG_4_T]_4_^6–^ (795 Å^2^). These five collision cross sections were measured in a helium drift tube mass spectrometer [34]. For the telomeric G-quadruplexes, collision activation upon entrance in the IMS cell is promoted by changing the trap DC bias from 18 to 30 V. The helium cell DC is 30.2 V and the IMS DC entrance is 7.3 V.

### Circular Dichroism (CD)

CD experiments were performed to obtain information on the DNA strands orientation. Experiments were run on a Jasco J-815 spectrophotometer using a quartz cell of 2 mm path length. All spectra shown here are the sum of three scans, acquired at 20°C with a scan speed of 50 nm/min and a 2 nm bandwidth. The CD data were normalized to molar circular-dichroic absorption (*Δε*) based on DNA concentration using the following Equation,

1 $$ \varDelta \varepsilon =\frac{\theta }{32980\times c\times l} $$

where θ is the CD ellipticity in milidegrees, *c* is DNA concentration in mol L^–1^ (5 × 10^–6^ mol.L^–1^), and *l* is the pathlength in cm (0.2 cm).

## Results and Discussions

### G-Quadruplexes from TMAA/KCl Solutions Show a Specific Number of K^+^ Adducts Corresponding to Coordination Between the G-Quartets

**Figure 2:**
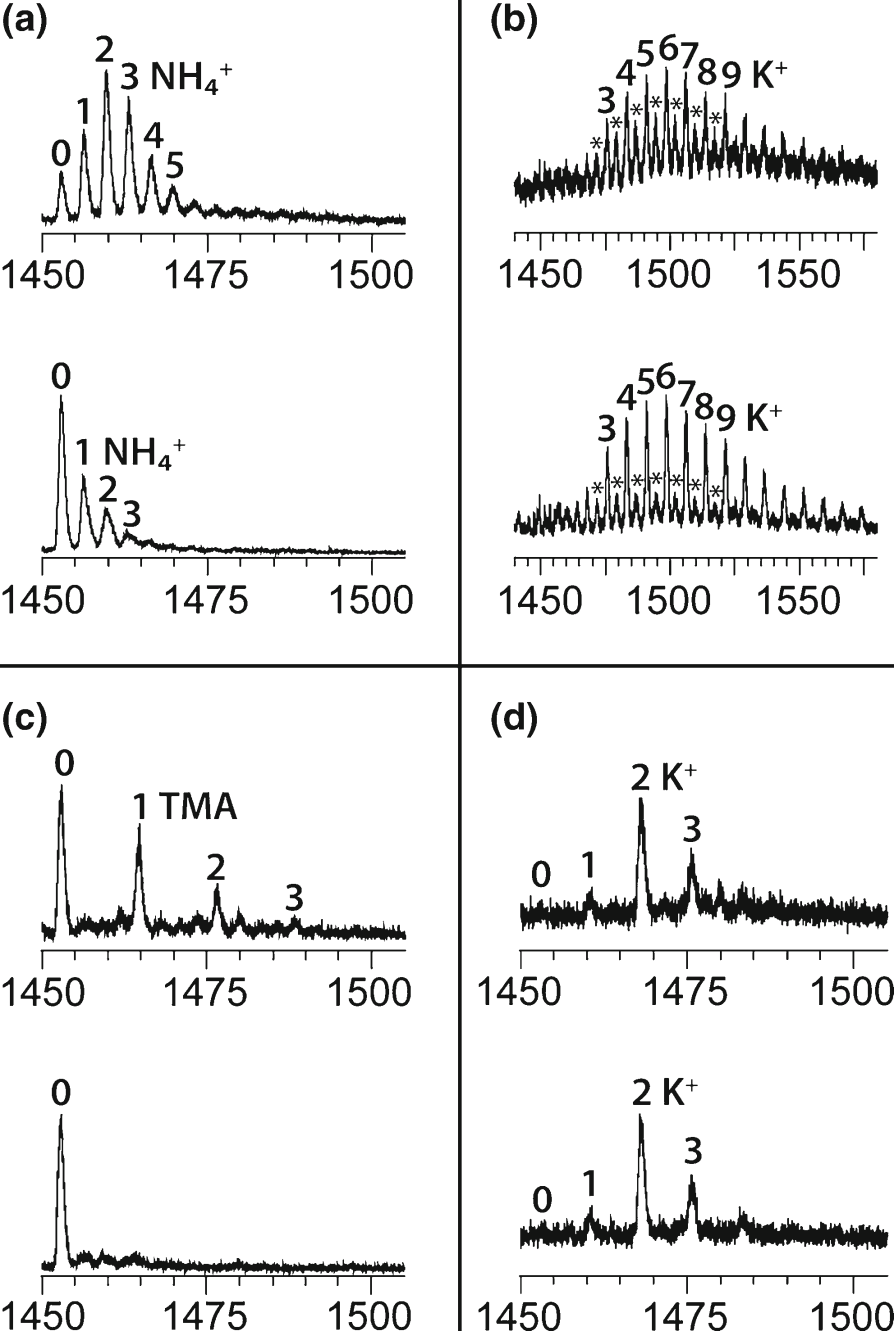
ESI-MS of 5 μM 23TAG into **(a)** 100 mM NH_4_OAc, **(b)** 100 mM NH_4_OAc + 1 mM KCl, **(c)** 100 mM TMAA, and **(d)** 100 mM TMAA + 1 mM KCl. The top mass spectra were recorded with an ion guide 1 voltage of 50 V and the bottom ones with 70 V. The annotations represent the number of adducts on the 5- charge state. The peak series with stars corresponds to mixed NH_4_^+^/K^+^ adducts

The mass spectrometer is tuned so that when the ion guide 1 is 50 V, the native structure is kept in the gas phase, as tested with the sensitive G-quadruplex [dG_4_T_4_G_4_]_2_ in 100 mM NH_4_OAc (Supporting information S1) [22]. Figure 2a shows a mass spectrum of the human telomeric sequence 23TAG in 100 mM NH_4_OAc in native conditions (low ion guide 1 voltage: 50 V) (Figure 2a, top). G-quadruplexes chelating two ammonium cations constitute the most abundant species, but a distribution of adducts is nevertheless observed (from 0 up to 5 or 6 ammonium cations). If the structure was unknown, it would actually be difficult to conclude on the preferred ammonium binding stoichiometry, as there is no clear bias in the ammonium ion distribution at any voltage. When the ion guide 1 voltage is increased from 50 to 70 V (Figure 1a, bottom) the complex is disrupted, the ammonium ions are released as NH_3_ and the most abundant species becomes the free oligonucleotide.

In order to examine the effect of adding potassium to the solution, we doped the ammonium acetate solution by 1 mM potassium chloride (Figure 2b). In these conditions, the resulting mass spectra are noisy at low *m/z* range (not shown) due to clusters formation, and there are numerous adducts on the G-quadruplexes peaks. As only two specifically bound K^+^ ions are anticipated, additional adducts are therefore nonspecific external adducts. Therefore, in ammonium acetate solutions doped with KCl, it is not possible to deduce the number of specifically bound potassium ions in the G-quadruplexes. Besides, two series of peaks are observed at low source voltage, indicating that each inter-quartet binding site may be occupied either by NH_4_^+^ or by K^+^.

To drive K^+^ ions to occupy inter-quartet sites specifically, we used trimethylammonium acetate (TMAA) as volatile buffer. ESI-MS recorded in TMAA alone show predominantly no TMA adduct (Figure 2c), in line with the fact that the trimethylammonium cation is too bulky to fit between G-quartets. At low ion guide 1 voltage (50 V, top) some TMA adducts can be observed but the shape of the distribution is typical of nonspecific adducts. This was confirmed with a control single strand incapable of forming a G-quadruplex in solution (Supporting Information S2). When the ion guide 1 voltage is set to 70 V (bottom) the only resulting peak is the DNA alone.

Figure 2d shows 23TAG electrosprayed in TMAA doped by 1 mM KCl. The most abundant species is the G-quadruplex chelating 2 potassium cations. This stoichiometry was expected because the structure adopted by 23TAG contains three G-quartets, and because K^+^ specific binding sites are located between quartets, there are two potassium binding sites per G-quadruplex. When the voltage is increased to 70 V, the remaining trimethylammonium (TMA) adducts are expelled, but the K^+^ adduct distribution remains identical, and clearly biased towards the 2-K^+^ adduct. The monovalent ion binding stoichiometry can be obtained with much more confidence in the TMAA/KCl conditions, and evidence of intramolecular G-quadruplex formation can be concluded with more confidence than in pure NH_4_OAc.

### TMAA Suppresses Nonspecific Alkali Adducts More Efficiently than NH_4_OAc, Including in Native Conditions

**Figure 3:**
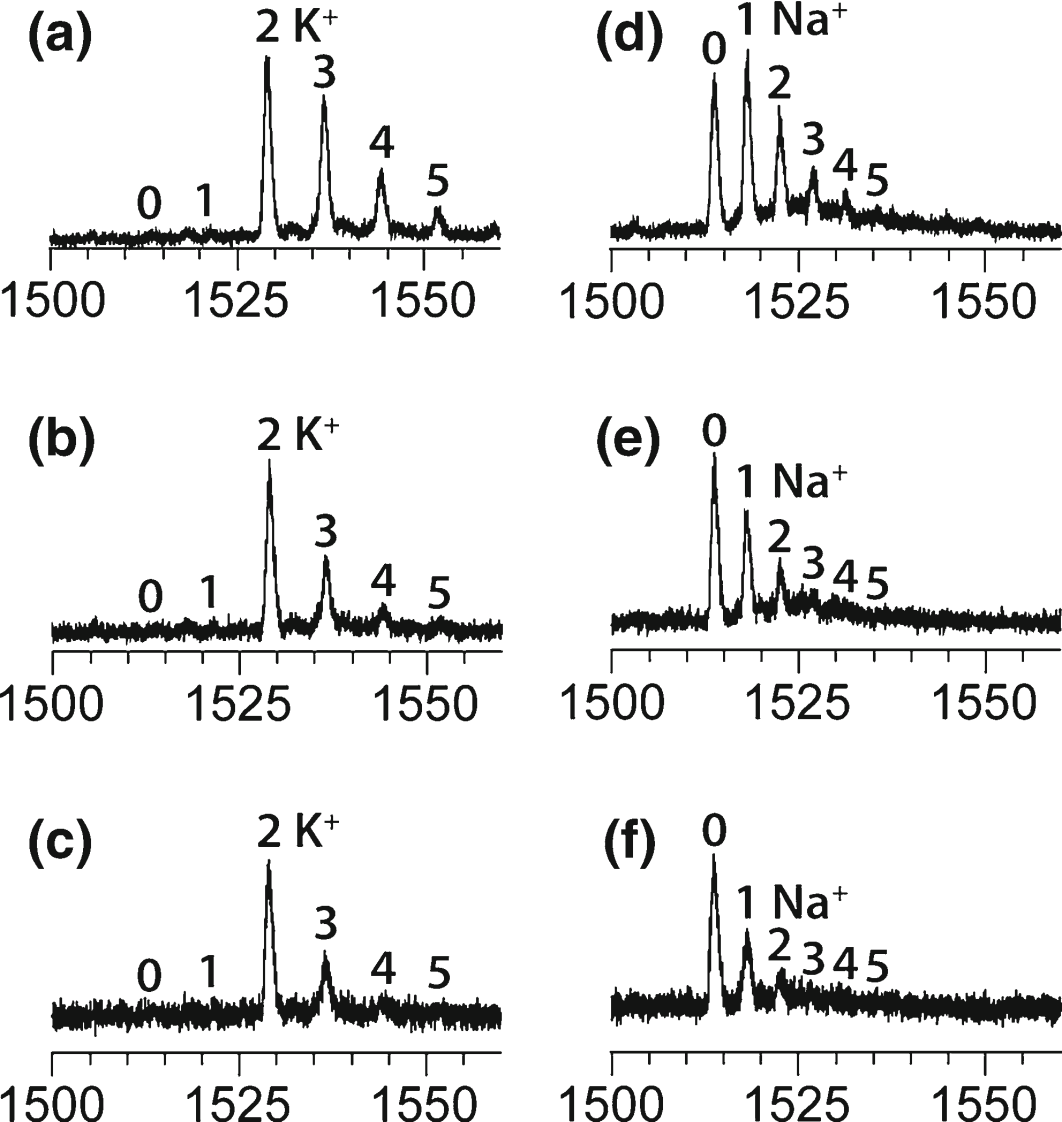
ESI-MS of 5 μM 24TTG into increasing amount of TMAA. Left: 1 mM KCl with **(a)** 50 mM, **(b)** 100 mM, and **(c)** 150 mM TMA, respectively. Right: 1 mM NaCl with **(d)** 50 mM, **(e)** 100 mM, and **(f)** 150 mM TMA, respectively. Mass spectra were recorded with an ion guide 1 voltage of 70 V. The annotations represent the potassium **(a)**–**(c)** or sodium **(d)**–**(f)** adducts on the 5- charge state

The comparison between Figure 2b and d illustrates that using TMAA instead of NH_4_OAc as electrolyte at pH = 7 allows the elimination of undesirable nonspecific K^+^ adducts. This may be due to the fact that the trimethylammonium ion has greater affinity than ammonium ion for the external nonspecific binding sites, so that it is more efficient in competing with alkali cations. To test that hypothesis, we investigated the effect of the TMAA concentration on potassium and sodium adducts in the 24TTG human telomeric sequence (Figure 3). For all tested TMAA concentration (50 mM, Figure 3a; 100 mM, Figure 3b, and 150 mM, Figure 3c) in the case of potassium the most abundant species is the G-quadruplex with two potassium cations. What changes is the number of additional, nonspecific K^+^ adducts. When TMAA concentration is increased, nonspecific adducts are increasingly suppressed to give almost exclusively the G-quadruplex with 2 potassium ions.

**Figure 4:**
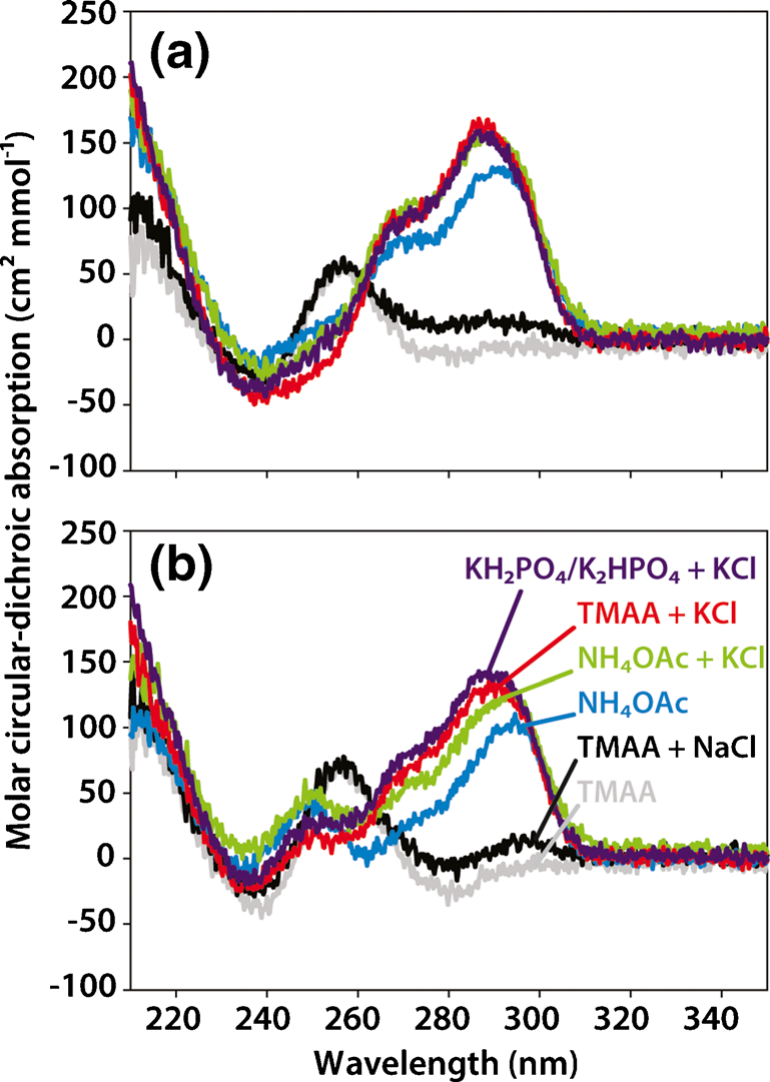
CD spectra of 5 μM 24TTG **(a)** and 23TAG **(b)** in different solution conditions (see also annotation in panel **(b)**: 20 mM KH_2_PO_4_/K_2_HPO_4_ + 70 mM KCl at pH 7 (purple), in 100 mM NH_4_OAc (blue), in 100 mM NH_4_OAc + 1 mM KCl (green), in 100 mM TMAA (grey), in 100 mM TMAA + 1 mM KCl (red), or in 100 mM TMAA + 1 mM NaCl (black)

In the case of sodium (Figure 3d, e, and f), the same effect was observed. However the final distribution is biased towards zero Na^+^ adduct, suggesting that no G-quadruplex is formed. This was confirmed by CD spectroscopy (Figure 4 and section below). The nonspecific adduct suppression effect was also confirmed with other DNA structures (see Supporting Information S3). The maximum KCl or NaCl concentration amenable to electrospray in 100 mM TMAA is about 1 mM. Above this concentration the signal/noise ratio decreases significantly and the formation of clusters at lower *m/z* becomes predominant. This concentration is sufficient for potassium, but not sodium, to induce the formation of the G-quadruplexes studied here. The lesser affinity of the G-quadruplexes for sodium than for potassium is well documented [35]. The remaining portion of the present paper focuses on G-quadruplexes formed in potassium conditions.

Besides displacing nonspecific adducts, increasing the TMAA concentration has also another effect: the charge states are displaced to lower values (Supporting Information S4). The lower charge states presented more numerous nonspecific adducts than the 5- presented here. Keeping the TMAA concentration at 100 mM allowed a good compromise to determine stoichiometry thanks to 5- charge state without losing too much signal. Triethylammonium acetate (TEAA) was also tested because it was reported to displace alkali ions even better than TMAA [28]. However, TEAA displaced totally the charge state 5- to 4-, and the adduct suppression effect of TEAA was less efficient on the 4- charge state than TMAA on the 5- charge state (data not shown). TMAA is the best compromise to obtain clean ESI mass spectra at low ion internal energies, which is important to preserve the gas-phase structure (see below).

### The G-Quadruplex Structures Formed in 100 mM TMAA + 1 mM KCl Are Similar to Those Formed in 100 mM KCl/Phosphate Buffer

To check whether the structures of the G-quadruplexes formed in TMAA/KCl are closer to those formed in pure NH_4_OAc or in pure KCl, circular dichroism (CD) spectroscopy was used. This technique is sensitive to the orientation of the strands in G-quadruplexes studies [36]: a maximum at 260 nm and a minimum at 240 nm indicate the presence of parallel G-quadruplex with homo-stacking between all-*anti* guanine quartets. A maximum at 290 nm and a minimum at 260 nm indicate the presence of antiparallel G-quadruplex with alternate *syn-anti* guanine stacking motifs. Hybrid G-quadruplexes will show two maximum peaks at 260 and 290 nm, as they possess both types of stacking. A mixture of structures in solution will result in a CD spectrum containing the weighted average of each contribution.

In Figure 4, we compare the CD spectra of the human telomeric DNA sequences in ammonium acetate, in phosphate buffer conditions (20 mM KH_2_PO_4_/K_2_HPO_4_ + 70 mM KCl, the buffer in which the NMR structures were determined), and in the different solution compositions tested here by ESI-MS (24TTG, Figure 4a and 23TAG, Figure 4b). The fact that the structures differ in KCl and in NH_4_OAc (Figure 4, purple and blue, respectively) was already known [20]. The structure of G-quadruplexes formed in ammonium acetate are generally intermediate between the ones formed in KCl (predominant in the nucleus) [37] and in NaCl [20].

In TMAA without KCl (Figure 4, grey), both DNA sequences adopt the same kind of CD spectra, characteristic of unfolded DNA. When 1 mM NaCl is added to TMAA (Figure 4, black), the species remains unfolded, confirming the anticipation from the absence of specific 2-Na^+^ adduct observed in the MS experiments in the same conditions (Figure 3e). However, when 1 mM KCl is added in the TMAA solution (Figure 4, red) the G-quadruplexes formed adopt almost exactly the same CD spectra as the ones in the phosphate buffer conditions. KCl-doped ammonium acetate solutions (Figure 4, green) showed CD spectra closer to the purely KCl form, but not as close as the KCl-doped TMAA conditions. In conclusion, the 100 mM TMAA/1 mM KCl conditions are compatible with electrospray mass spectrometry and favor the formation of structures that possess almost identical proportion of homo-stacking versus alternate stacking as in 100 mM K^+^ solutions. Further studies, for example by NMR in deuterated TMAA, will be needed to determine if the structures are indeed identical.

### Ion Mobility Spectrometry Experiments Indicate a Similar Shape for Telomeric G-Quadruplexes Preserving Two K^+^ or NH_4_^+^

**Figure 5:**
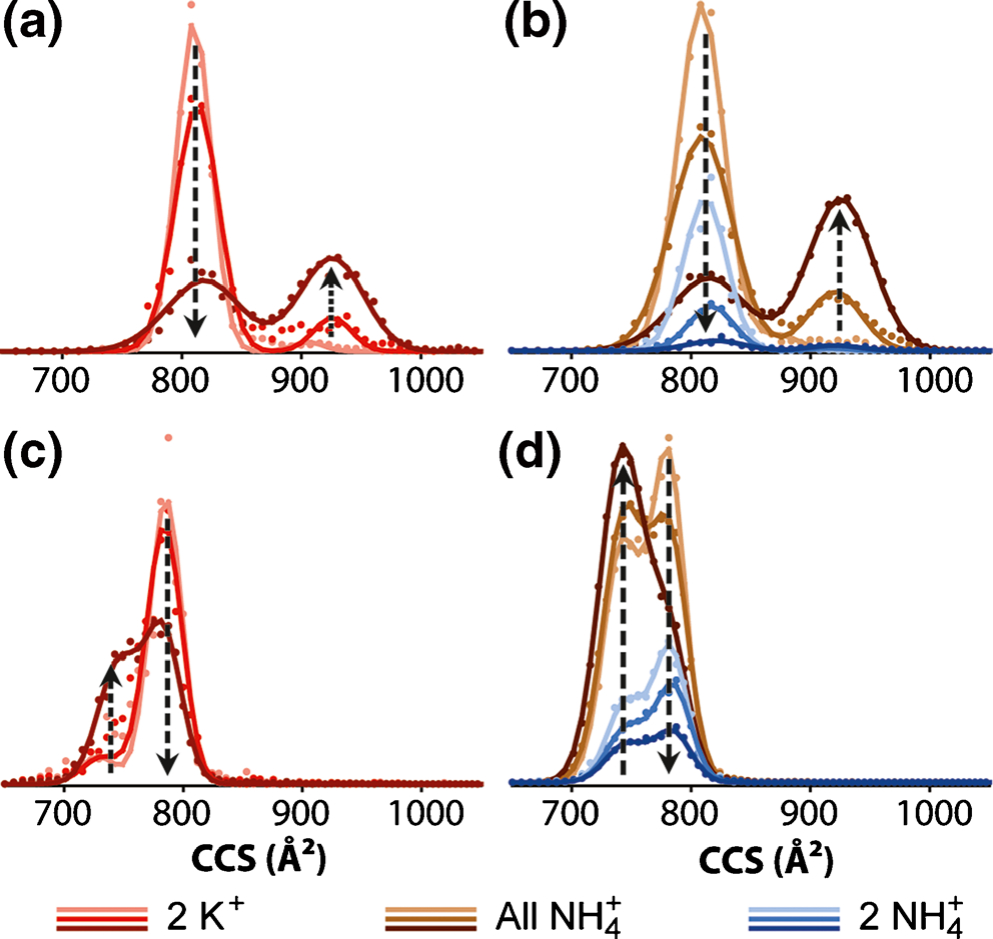
Collision cross section distribution of 5 μM 24TTG into 100 mM TMAA + 1 mM KCl **(a)** and **(c)** or 100 mM NH_4_OAc **(b)** and **(d). (a)** and **(b)** represent the 6- ions and **(c)** and **(d)** the 5- ions. Bias voltage increases from light to dark colors (18, 25, and 30 V, respectively). Brown colors represent the collision cross section reconstructed from the sum of the G-quadruplexes with zero, one, and two ammonium cations and blue ones are the collision cross section of the G-quadruplexes with two ammonium ions exclusively. Red colors are the G-quadruplexes with two potassium ions

In MS experiments on G-quadruplex in NH_4_OAc, ammonium ion preservation in the gas phase structure is believed to ensure the preservation of the solution-phase structure [22, 38]. Is that the same in the case of K^+^ ions? To probe the gas-phase structure of the G-quadruplexes, we used ion mobility spectrometry coupled to mass spectrometry (IMS-MS). This experiment links the arrival time of an ion inside the mobility cell to a shape factor called collision cross section (CCS). Figure 5 shows the results: two conditions are compared, 100 mM TMAA + 1 mM KCl (red) and 100 mM NH_4_OAc (brown and blue) for the sequence 24TTG. Very similar results were obtained for 23TAG (Supporting Information S5). The top graphs show the results for the 6- charge state [TMAA/KCl in (A), NH_4_OAc in (B)] and the bottom graphs are for the 5- ions. The bias voltage right before the mobility cell entrance was varied (18 V, 25 V, or 30 V). The NH_4_^+^-binding stoichiometries vary with the bias voltage, in a similar way as they vary with source voltages (see Figure 2 and discussion thereof). For that reason, we represented the integrated CCS of all adduct species (0, 1, and 2 NH_4_^+^ adducts summed, in brown, and the CCS integrated only over the mass spectral peak corresponding to two NH_4_^+^ ions bound (blue). CCS distributions were normalized to their total area in order to reflect the proportion of folded or unfolded G-quadruplexes with two ammonium ions.

At low bias voltage (18 V) the CCSs were similar in both conditions: one peak around 800 Å^2^ for 24TTG for the 6- ions (Figure 5a and b). For the 5- ions (Figure 5c and d) the CCS peak appears at smaller values; which is typical in ion mobility spectrometry because the overall structure is less charged and then less dilated. In native conditions, the structures are very close in term of CCS in NH_4_^+^ (light brown) or K^+^ (light red). Interestingly, the Gaussian curve that fits the data is a little bit broader in ammonium ion conditions. This can be due to a greater floppiness or to a greater polymorphism in ammonium ion solution. When the bias voltage increases (from light to dark colors) the peaks at the initial CCS value decrease and at the expense of peaks at higher CCS (for 6- charge state) or lower CCS (for 5- charge state). Expansion of high charge states and compaction of lower charge states upon energy increase was noted for other G-quadruplexes in ammonium conditions [38].

In the case of ammonium ions, the gas-phase structure is changing when the voltage is increased, and ammonia loss observed in the mass spectra gives a hint that denaturation happens in the gas phase. When two potassium ions are trapped inside the G-quadruplex formed from TMAA/KCl, the collisional activation has the same unfolding effect, although the potassium ion adducts are not lost. Therefore, although the voltage at which unfolding occurs is higher in the presence of potassium than ammonium, gas-phase unfolding still occurs. Importantly, these changes in ATD are not accompanied by visible changes in the mass spectra for the TMAA/KCl conditions: the K^+^ ions cannot escape from the multiply charged anions.

### The KCl Concentration Required for G-Quadruplex Formation in TMAA Depends on the Sequence

**Figure 6:**
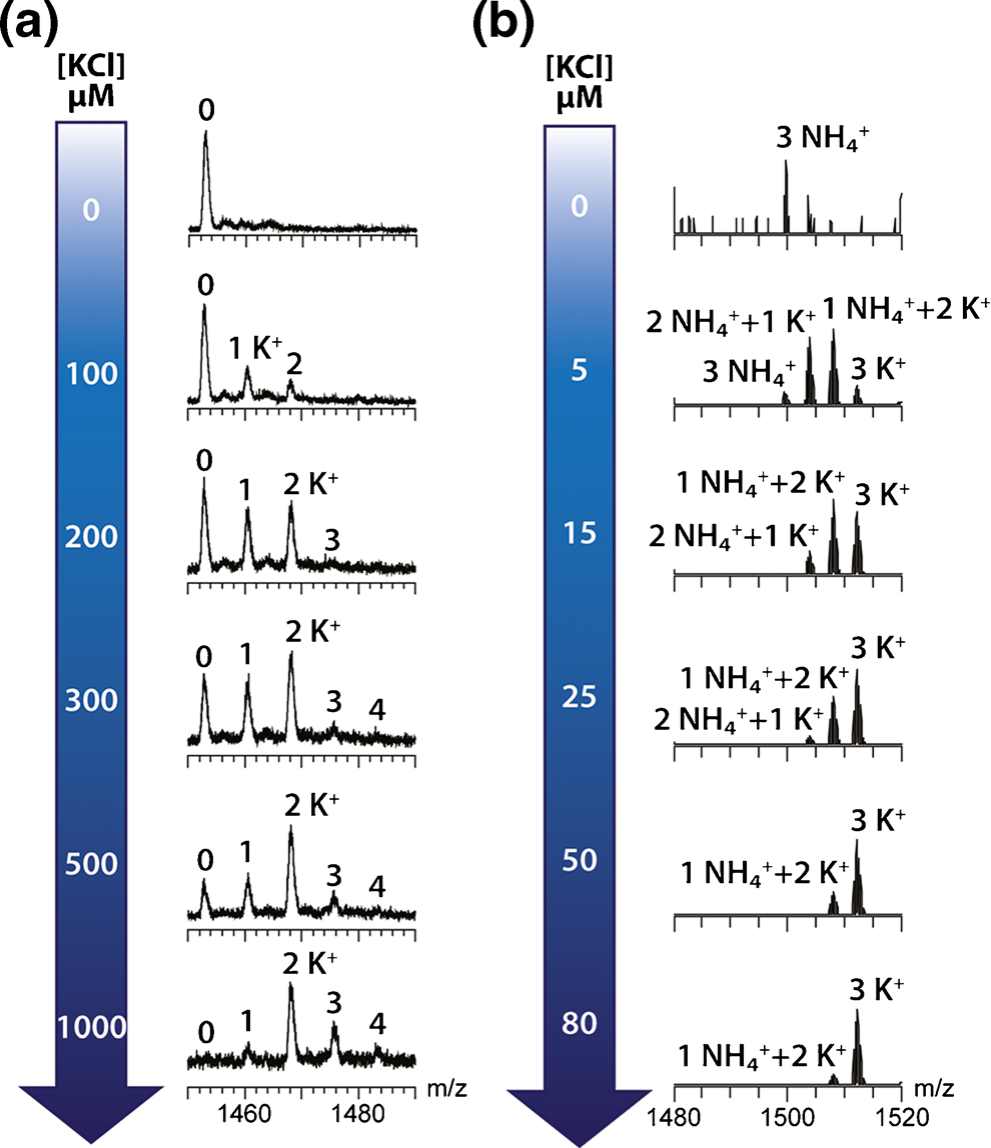
KCl titration of **(a)** 5 μM 23TAG and **(b)** 20 μM TG_4_T single strand into 100 mM TMAA. The annotations indicate the number of adducts on the 5- charge state. In titration **(a)**, the single strand was allowed to react for 4 h at room temperature before the mass spectra were recorded using the LCT mass spectrometer. In titration **(b)**, the solutions were prepared with 100 μM in TG_4_T single strand for 24 h before being diluted to 20 μM, and the mass spectra were recorded on the Exactive mass spectrometer

Above, we showed results with the maximum concentration we could electrospray while keeping clean mass spectra (1 mM). Here we describe KCl titration experiments that probe the minimum KCl concentration required to fold the G-quadruplexes. Figure 6 shows two KCl titrations in 100 mM TMAA for the intramolecular telomeric sequence 23TAG (Figure 6a) and for the tetramolecular G-quadruplex [dTG_4_T]_4_ (Figure 6b). At 100 μM KCl, 23TAG G-quadruplexes are not formed: only a very small amount of potassium adducts are observed. When the KCl concentration is increased, the adduct distribution is shifted towards two potassium ions bound. Mass spectra at 200 and 300 μM unambiguously demonstrate cooperativity in K^+^ binding: the cation adducts distribution is non-statistical and depleted in 1-K^+^ adduct. This is very important to understand the folding mechanism of G-quadruplexes, and mass spectrometry experiments allow to unambiguously visualize the concerted incorporation of two potassium ions.

The second example is the titration of the single strand dTG_4_T, which forms a tetramolecular G-quadruplex with three coordinated monovalent ions. The titration is made on a lower concentrations range, as the G-quadruplex formation is already complete at 80 μM in KCl. Despite the fact that ammonium ions were not added intentionally, some ammonium adducts appear here at low KCl concentrations, and mixed NH_4_^+^/K^+^ adducts appear at intermediate KCl concentrations (the sum of adducts always being three cations, i.e., one between each G-quartet). Ammonium traces come from the oligonucleotide synthesis and, therefore, traces of ammonium-ion bound tetramers are preformed in the lyophilized oligonucleotides. However the single strand is prevalent (see full scale mass spectra in the Supporting Information S6).

The mechanism of formation of the tetramolecular G-quadruplex formation is very different from the formation of the intramolecular quadruplex. Adding a very small amount of potassium into the solution induced the cooperative tetramerization of TG_4_T. To be stabilized, the tetramer must always contain total of three cations in its central channel. Then, because potassium has a higher affinity for G-quadruplexes than ammonium, the equilibrium is displaced specifically towards the 3-K^+^ adduct by addition of higher KCl amounts. This example further illustrates the correlation between the observation of *n* K^+^ adducts and the presence of *n + 1* G-quartets [21].

## Conclusions

We reported here for the first time that by using trimethylammonium acetate (TMAA) as electrolyte, electrospray mass spectra of G-quadruplexes can be obtained in the presence of the likely physiologically relevant potassium cation. Moreover, circular dichroism experiments indicate that the guanine stacking motifs and, therefore, relative strand orientations are very similar in 100 mM TMAA/KCl and 100 mM KCl, so the presence of TMAA in the ESI-MS compatible solution does not affect the overall structure of the DNA G-quadruplexes studied here. This is particularly important for polymorphic G-quadruplexes such as those formed by the human telomeric sequence. Also, our conditions make it easier to determine the number of potassium ions specifically coordinated to the structure and, hence, to ascertain the presence of a G-quadruplex in solution and how many G-quartets are involved.

The perspectives open by the present work are numerous. First, as in TMAA/KCl solutions the K^+^-binding stoichiometry is determined easily. In NH_4_OAc conditions, the ESI-MS determination of cation binding stoichiometry was clear only for parallel G-quadruplexes, which robustly preserved their ammonium ions in the gas phase, whereas the antiparallel structures did not. The new method can be used to probe the number of specific binding sites in other sequences, for example issued from bioinformatics studies. Second, as TMAA allows spraying potassium-bound G-quadruplexes in soft conditions, this opens the way to probing preserved structures in the gas phase, as shown by our preliminary experiments with ion mobility spectrometry. Third, the TMAA/KCl conditions are interesting for ligand screening, in order to test the binding stoichiometry and affinity for potassium-bound structures. ESI-MS can also be used to understand the G-quadruplex folding mechanism and, more specifically, the interplay between cation binding and folding. Detailed investigation of the folding of various human telomeric sequences upon K^+^ or ligand titration is currently under way in our laboratory. Finally, it would be interesting to test whether the use of TMAA can be extended to other systems than DNA G-quadruplexes, for example to RNA-magnesium complexes, or to protein complexes.

## Electronic supplementary material

Below is the link to the electronic supplementary material.

ESM 1 (PDF 424 kb)
