# Supplementary material for: Native Electrospray Mass Spectrometry of DNA G-Quadruplexes in Potassium Solution
Source: J Am Soc Mass Spectrom. 2014 Apr 30;25(7):1146–54. doi: 10.1007/s13361-014-0890-3 (PMC4055847; doi:10.1007/s13361-014-0890-3)
Supplement: Supplementary file 1 — (PDF 424 kb) [file 13361_2014_890_MOESM1_ESM.pdf]

# Native electrospray mass spectrometry of DNA G-quadruplexes in potassium solution

A. Marchand[1,2], V. Gabelica[1,2,*]

[1] Univ. Bordeaux, IECB, ARNA Laboratory, F-33600 Pessac, France.

[2] Inserm, U869, ARNA Laboratory, F-33000 Bordeaux, France.

* Corresponding author email: valerie.gabelica@inserm.fr

## SUPPORTING INFORMATION

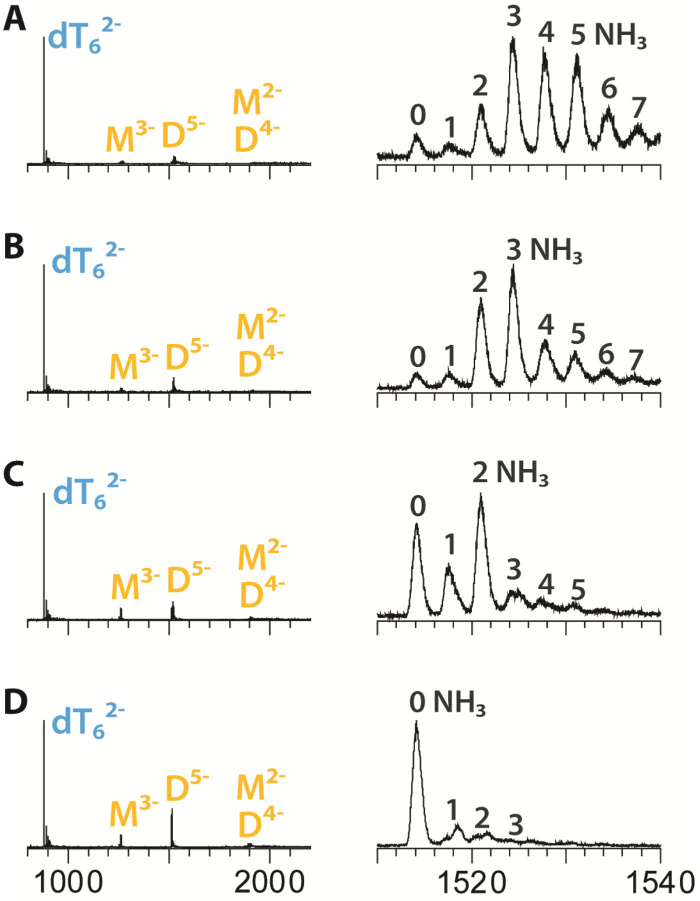


**Figure S1.** ESI-MS of 10 µM $G_4T_4G_4$ in 100 mM $NH_4OAc$. From A to D (30, 50, 70 and 90 V, respectively): the effect of the ion guide 1 voltage on the ammonium cation preservation in the gas-phase. Left is the full scale spectrum and on the right is the zoom on the dimer $D^{5-}$ peak.

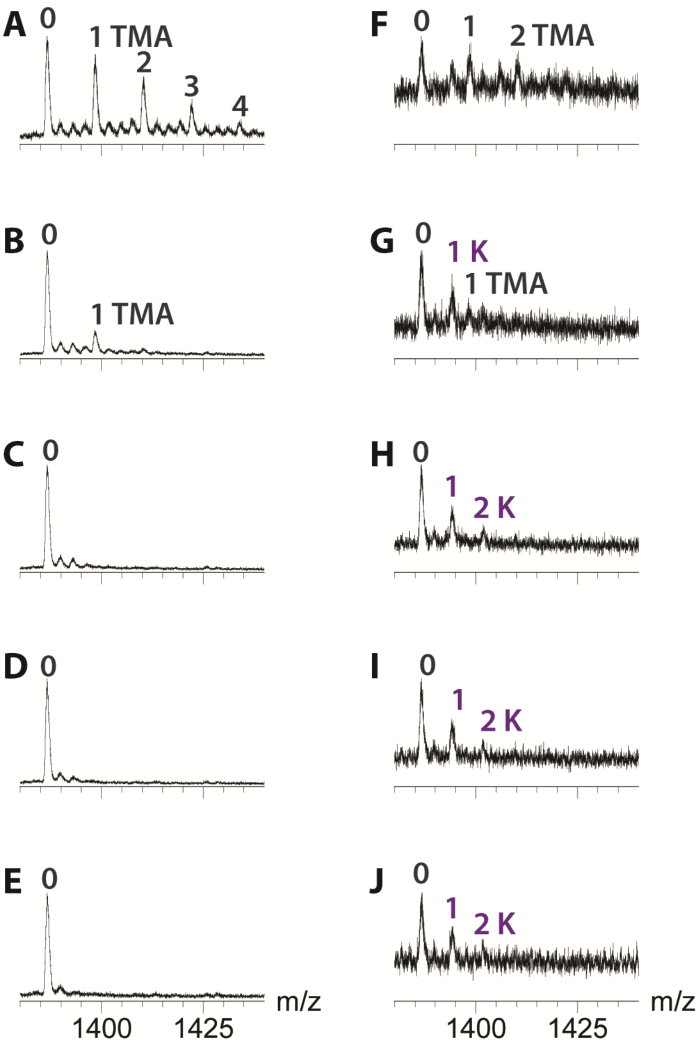


**Figure S2.** ESI-MS of 5 μM 22non-G4 sequence d(GGG-ATG-CGA-CAG-AGA-GGA-CGG-G). From A to E and from F to J the ion guide 1 voltage is increased to 30, 50, 70, 90 and 110 V, respectively. (A-E): 22non-G4 in 100 mM TMAA and (F-J): 22non-G4 in 100 mM TMA + 1 mM KCl. The predominant stoichiometry is the oligonucleotide without any adducts.

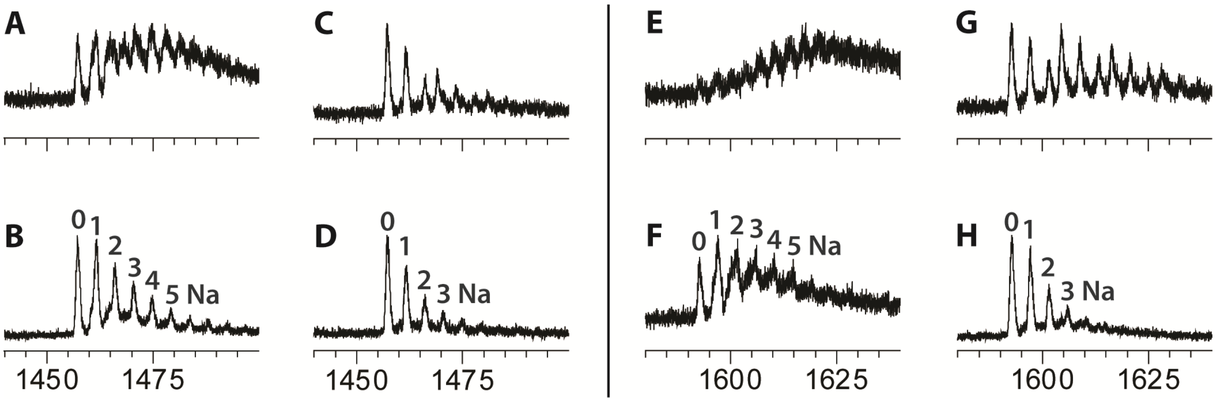


**Figure S3.** ESI-MS of 5 µM duplex DNA made by two d(CGC-GAA-TTC-GCG) strands = DK66 (A-D) and 5 µM hairpin DNA d(CAA-TCG-GAT-CGA-ATT-CGA-TCC-GAT-TG) hp26 (E-H) in 100 mM $NH_4OAc$ + 1mM NaCl (A-B and E-F) or 100 mM TMAA + 1 mM NaCl (C-D and G-H). (A, C, E and G) Ion guide 1 voltage is 50 V and (B, D, F and H) 70 V. Zoom on the 5- charge state. It appears clearly that TMAA is more efficient to decrease the amount of nonspecific adducts.

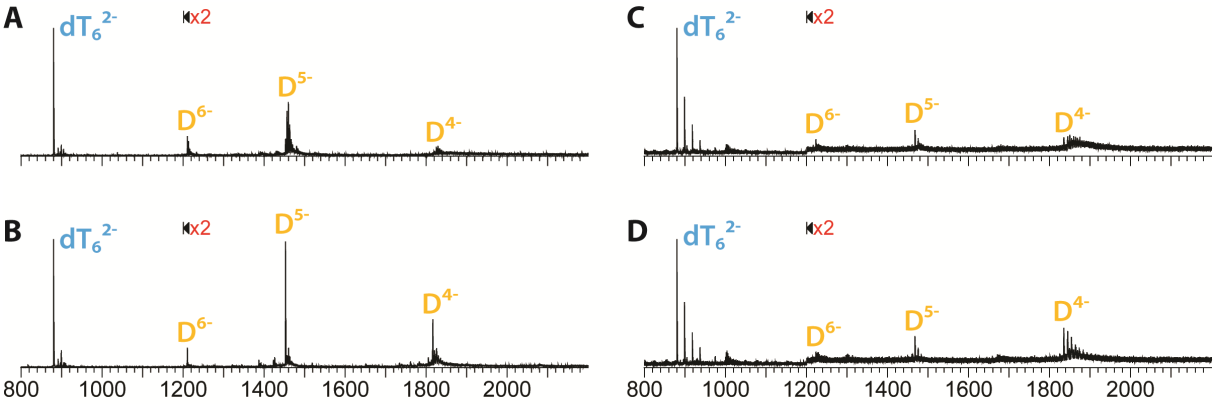


**Figure S4.** ESI-MS of 5 µM 23TAG; (A) and (B): electrosprayed in 100 mM $NH_4OAc$ with ion guide 1 of 50 and 70 V, respectively; (C) and (D) in 100 mM TMAA + 1 mM KCl with ion guide 1 of 50 and 70 V, respectively. Note the 2× zoom in the 1200 – 2200 m/z region. In $NH_4OAc$, charge states are clearly centered on the 5- form. In TMAA + KCl at 70 V the 4- becomes the predominant charge state.

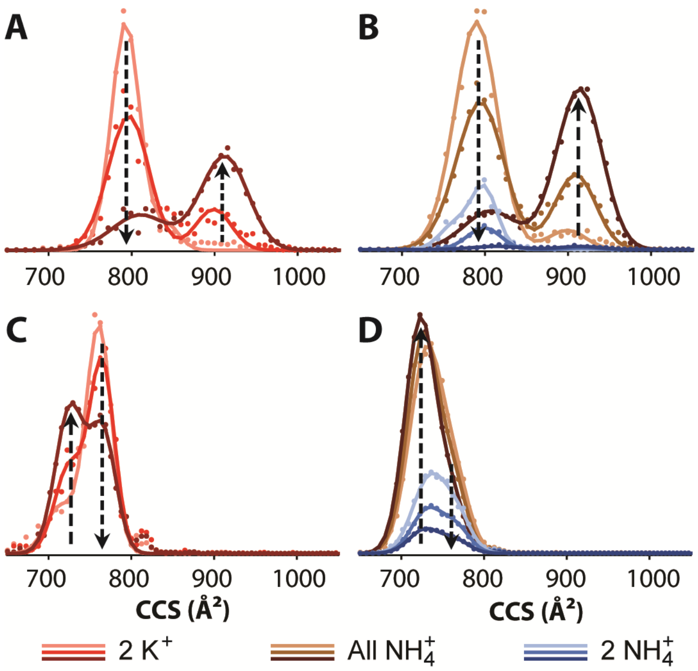


**Figure S5.** Collision cross section distribution of 5 µM 23TAG in 100 mM TMAA + 1mM KCl (red) or 100 mM $NH_4OAc$ (brown and blue). Bias voltage increase from light to dark colors (respectively 18, 25 and 30 V). Brown colors represent the collision cross section reconstructed from the sum of the G-quadruplexes with zero, one and two ammonium cations and blue ones are the collision cross section of the G-quadruplexes with two ammonium ions exclusively. Red colors are the G-quadruplexes with two potassium ions.

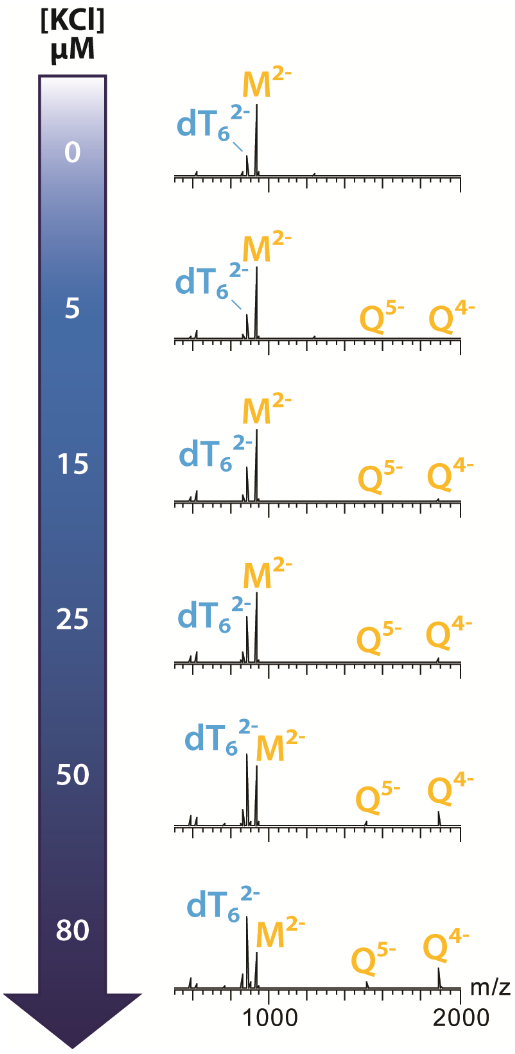


**Figure S6.** Mass spectrometry titration of 20 µM $TG_4T$ into 100 mM TMAA doped by KCl. Full scale ESI-MS are showed. M and Q represent the monomer and quadruplex species, respectively. The solutions were prepared with 100 µM in $TG_4T$ single strand for 24 hours before being diluted to 20 µM.
